# Current diagnosis and treatment practice of central retinal artery occlusion: results from a survey among German stroke units

**DOI:** 10.1186/s42466-022-00193-w

**Published:** 2022-08-01

**Authors:** Carolin Hoyer, Simon Winzer, Egbert Matthé, Ida Heinle, Vesile Sandikci, Darius Nabavi, Michael Platten, Volker Puetz, Kristina Szabo

**Affiliations:** 1 Department of Neurology and Mannheim Center for Translational Neuroscience, Medical Faculty Mannheim, Heidelberg University, Theodor-Kutzer-Ufer 1-3, 68167 Mannheim, Germany; 2 Department of Neurology, Carl Gustav Carus University Hospital, Technische Universität Dresden, Dresden, Germany; 3 Dresden Neurovascular Center, Carl Gustav Carus University Hospital, Technische Universität Dresden, Dresden, Germany; 4 Department of Ophthalmology, Carl Gustav Carus University Hospital, Technische Universität Dresden, Dresden, Germany; 5 Department of Neurology, Vivantes Klinikum Neukölln, Berlin, Germany

**Keywords:** Acute stroke therapy, Ischemic stroke, Neurology, rtPA, Stroke units, Treatment

## Abstract

**Background:**

Central retinal artery occlusion (CRAO) is a neuro-ophthalmological emergency whose optimal management is still under debate and due to the absence of definite guidelines, practice is expected to vary. We aimed to characterize early evaluation as well as acute treatment and diagnostic approaches in German hospitals with a stroke unit (SU).

**Methods:**

In 07/2021, all 335 certified German SUs were invited to participate in an anonymous online survey endorsed by the German Stroke Society on emergency department care organization, diagnostic procedures, and treatment of patients with unilateral vision loss (UVL) subsequently diagnosed with CRAO.

**Results:**

One hundred and sixty-three (48.6%) of the 335 eligible centers responded. Most (117/135; 86.7%) stated that UVL patients were treated as an emergency, in 62/138 (44.9%) hospitals according to specific guidelines. First-line evaluation was performed by neurologists in 85/136 (62.5%) hospitals, by ophthalmologists in 43/136 (31.6%) hospitals. Seventy of 135 (51.9%) respondents indicated a lack of on-site ophthalmological expertise. Seventy-four of 129 (57.4%) respondents performed thrombolysis in CRAO and 92/97 (94.8%) stated that patients with CRAO–if admitted to neurology–were treated on a SU.

**Conclusions:**

Our findings reflect notable heterogeneity in early intrahospital care of CRAO in German SUs but demonstrate a preference for work-up and management as acute stroke by the involved neurologists. Streamlining interdisciplinary emergency evaluation is essential for ongoing and future prospective trials.

**Supplementary Information:**

The online version contains supplementary material available at 10.1186/s42466-022-00193-w.

## Introduction

Central retinal artery occlusion (CRAO) as one of the causes underlying painless acute monocular loss of vision constitutes a neuro-ophthalmological emergency. It yearly affects about 1 in 100.000 people with a slight male preponderance [1, 2] and is etiologically heterogeneous with a variety of associated local and systemic disorders. CRAO leads to substantial permanent impairment of eyesight or even blindness in the vast majority of patients due to the low threshold for irreversible retinal damage under conditions of ischemia [3, 4]. The pathomechanism most frequently implicated in CRAO is thromboembolic occlusion of the central retinal artery from e. g. large vessel disease of the carotid artery or of cardiac origin. Hence, CRAO shares a common etiological denominator with cerebral ischemic stroke and has accordingly been recognized as a manifestation thereof by international neurological and ophthalmological societies [5], 2016; [6]. This notion is supported by a large body of data pertaining to imaging findings, clinical course and outcome: In a large portion of patients with CRAO, clinically silent infarcts are discernable on MRI [7, 8] and risk of incident stroke is almost 30-fold increased in the week following CRAO [9]. In addition, CRAO is an important indicator for vascular morbidity and mortality, in part mediated by the burden of vascular risk factors which also impact on the risk for cerebral ischemic stroke [10]. These aspects underscore the relevance of, first, the swift and correct identification of CRAO patients and, second, adequate work-up and management regarding the prophylaxis and treatment of risk factors and complications. Apart from this, there exists neither high-class evidence for, nor consensus on, any of the numerous treatments aimed at restoring visual acuity after CRAO. Rapid reperfusion of the retina represents a logical approach to treatment, and the existing data indicate that thrombolysis may be beneficial if initiated within 4.5 h after symptom onset [11]. However, we are still awaiting data from randomized controlled trials currently underway or soon to be recruiting [12, 13]. In light of the absence of definite treatment guidelines for CRAO, current practice is expected to be based on local experience and infrastructure and accordingly to vary to some extent among different hospitals. We aimed to assess the management practices of diagnosis and treatment of CRAO patients in German hospitals with a stroke unit (SU) with focus on emergency evaluation conducted by neurologists as well as acute diagnostic and therapeutic measures.

## Methods

In July 2021, all 335 certified SUs in Germany received an email invite by the German Stroke Society to participate in a standardized anonymous online survey. The questionnaire was drafted and reviewed by KS, CH, VP, SW and EM. The survey assessed aspects relating to the organization of emergency department care (four questions), emergent diagnostic (two questions) and therapeutic procedures (six questions) as well as work-up performed in patients presenting with unilateral vision loss (UVL) and subsequently diagnosed with CRAO (four questions). In addition, the estimated numbers of CRAO patients seen and treated (six questions) and information regarding the structure and staffing of the emergency department (six questions) were solicited, one question assessed the willingness to participate in a clinical trial evaluating safety and efficacy of intravenous thrombolysis in acute CRAO. The option to comment in freeform text was provided at the end of the questionnaire. The questionnaire is provided as Additional file 1. As no patient data were requested, Institutional Review Board approval was waived.

## Results

Of the 335 certified German Stroke Units, 163 (48.6%) participated in the online survey. The questionnaire was fully completed by 118 (72.4%) of all respondents, respective hospital information is summarized in Table 1. The majority of responding institutions were non-academic hospitals (64%), had interdisciplinary emergency departments (58%), were certified as regional SUs (39%), and had a total of five to nine SU beds (40%).

**Table 1:** Responder characteristics

Variable	Percentage of responders (%)
*Type of hospital*	
Academic hospital	11.0
Non-academic hospital	64.4
No answer provided	24.6
*Organisation of ED*	
Interdisciplinary	57.7
Internal medicine	6.7
Specialty-based	8.6
Other	2.5
No answer provided	24.5
*Type of stroke unit*	
Regional	39.3
Cross-regional	35.6
Telemedicine*	0.6
No answer provided	24.5
*Number of stroke unit beds*	
1–4	3.7
5–9	40.5
10–14	22.7
15–19	5.5
20–24	1.8
> 24	1.2
No answer provided	24.6

Total number of responders = 163. All categories add up to 100%

*Neurological expertise provided within a telemedicine network

**Fig. 1:**
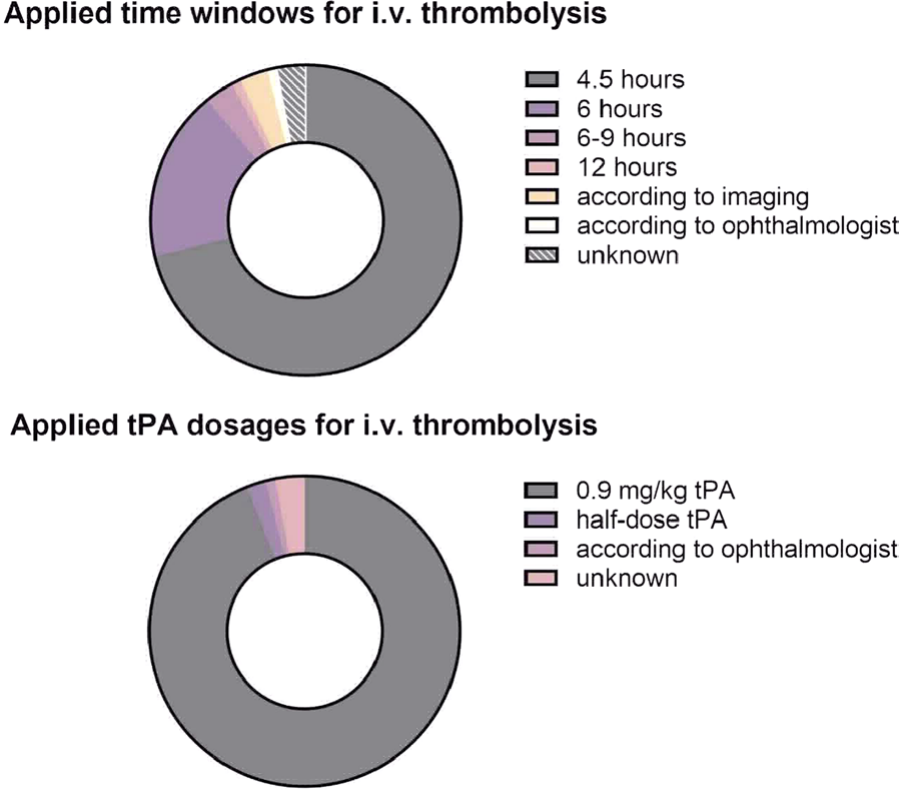
Differences in time-window for treatment and tPA dosage for intravenous thrombolysis in in patients with central retinal arterial occlusion as applied by the responders. tPA: tissue plasminogen activator

Most respondents (117/135; 86.7%) stated that patients with UVL are treated as an emergency in their hospital, however, a specific hospital guideline exists in only 62/138 (44.9%) centers. First-line evaluation is performed by neurologists in 85/136 (62.5%) hospitals and by ophthalmologists in 43/136 (31.6%) hospitals. Seventy respondents (51.9%) indicated a lack of on-site ophthalmological expertise. Fundoscopy is the most commonly reported diagnostic tool performed by the ophthalmologist in 59/135 (43.7%) hospitals, while a non-contrast CT is the most commonly initiated examination by the neurologist according to 119/138 (86.2%) responses. The replies suggest that neurologists request an ophthalmological consultation more often (81/135; 60.0%) than vice versa (51/135; 37.8%). In patients with CRAO, ocular massage is the most frequently performed ophthalmological treatment (29/129; 15.5%), while intravenous thrombolysis is regularly performed in suitable patients in 74/129 (57.4%) hospitals (Fig. 1). Intra-arterial thrombolysis is performed only in a minority of hospitals (4.7%). Ninety-two of 97 (94.8%) respondents stated that—if admitted to neurology—patients with CRAO are routinely or exclusively treated on the SU. If admitted to a SU, the vast majority of patients receive diagnostic evaluation in the manner of a dedicated stroke work-up, including extracranial ultrasound (121/128; 94.5%), transcranial ultrasound (119/128; 93.0%), ECG monitoring (113/128; 88.3%), as well as transthoracic (102/128; 79.7%) and transesophageal echocardiography (68/128; 53.1%). Similarly, medical secondary prevention in CRAO patients is initiated as indicated by stroke guidelines. For a summary of all replies, see Table 2. In a field for comments at the end of the survey, 15 respondents specified the problem that ophthalmological expertise was not available or not available in a timely manner at their institution. Of 91 institutions responding, 70 estimated the number of CRAO patients treated in neurology to be between 0 and 20 within the last two years.

**Table 2:** Details of diagnostic procedures and treatment of patients presenting with unilateral vision loss subsequently diagnosed with central retinal artery occlusion

Variable	Rate of positive responders, N (%)
*Emergency management*	
Patients with UVL assessed in ED	117/135 (86.7)
Specific hospital guideline available	62/138 (44.9)
First-line evaluation by ophthalmologists	43/136 (31.6)
First-line evaluation by neurologists	85/136 (62.5)
Lack of ophthalmological expertise / department	70/135 (51.9)
*Ophthalmological emergency assessment*	
Fundoscopy	59/135 (43.7)
Spectral-domain optical coherence tomography	11/135 (8.1)
Neurology consultation	51/135 (37.8)
*Neurological emergency assessment*	
Ophthalmology consultation	81/135 (60.0)
Computed tomography	119/138 (86.2)
Computed tomography angiography	109/135 (80.7)
Magnetic resonance imaging	26/135 (19.3)
Magnetic resonance angiography	26/135 (19.3)
Carotid ultrasound	100/135 (74.1)
Erythrocyte sedimentation rate	101/135 (74.8)
*Ophthalmological treatment*	
Ocular massage	20/129 (15.5)
Hyperbaric oxygen treatment	0/129 (0.0)
Paracentesis	3/129 (2.3)
Acetazolamid	6/129 (4.7)
Isovolemic hemodilution	13/129 (10.1)
*Neurological treatment*	
Intravenous thrombolysis	74/129 (57.4)
Intraarterial thrombolysis	6/129 (4.7)
In-patient treatment neurology	97/128 (75.8)
Admission to a stroke unit	92/97 (94.8)
*Stroke work-up*	
Extracranial ultrasound	121/128 (94.5)
Transcranial ultrasound	119/128 (93.0)
ECG monitoring	113/128 (88.3)
Transthoracic echocardiography	102/128 (79.7)
Transoesophageal echocardiography	68/128 (53.1)
*Secondary prevention*	
Antiplatelet therapy	121/128 (94.5)
Anticoagulation	116/128 (90.6)
Statin therapy	114/128 (89.1)

*UVL* Unilateral vision loss, *ED* emergency department, *ECG* electrocardiogram

## Discussion

Our survey focused on current diagnostic and therapeutic practices regarding CRAO in German hospitals with SUs. We found a considerable degree of variability in the management of CRAO patients. This is not surprising because an accepted treatment standard backed by high-quality evidence is still lacking, and the existence and application of in-house guidelines is not consistently in place. Patients presenting with UVL are allocated to the emergency department in most cases—hence, the urgent character of this symptom, warranting timely assessment, is immediately recognized upon hospital arrival. This, however, stands in pronounced contrast to the apparent lack of public awareness of vision impairment as a potential stroke symptom requiring urgent attention [14, 15] and knowledge of CRAO [16]. As a consequence, CRAO patients frequently present with considerable latency from symptom onset after an initial prehospital ophthalmology consultation [17, 18]. Moreover, diagnosis of CRAO is challenging for many emergency physicians [19], and fundoscopy, a skill requiring extensive training and constant maintenance to be correctly performed and interpreted, is infrequently employed by non-ophthalmologists, including neurologists [20]. Hence, early expert in-hospital ophthalmological examination is an essential component of a comprehensive assessment of patients with UVL. Nonmydriatic ocular fundus photography has been demonstrated to be feasible as an auxiliary tool in the emergency department including the diagnosis of retinal ischemia [21, 22], with further technological advances likely to improve usability in various contexts [23]. Alternatively, innovations in the field of teleophthalmology [24] may allow for fundus image acquisition in the ambulance, thus enabling expert ophthalmology input at a very early stage of the patient journey. More than half of the respondents in our survey indicated that they did not have on-site ophthalmological expertise. Thus, the correct disposition of patients with acute UVL to hospitals providing ophthalmological assessment and neurological stroke-unit care is of paramount importance. Moreover, increasing broad public and healthcare provider awareness regarding acute painless UVL as a manifestation of stroke should be established in order to optimize currently prevailing presenting and referral patterns [25]. The modification of mnemonics and screening tools to draw attention to and include visual impairment as a stroke symptom has shown some effect in reducing the number of missed strokes including patients presenting with visual disturbance [26].

The natural history of visual outcome in CRAO is highly variable and depends on the subtype of disease: in non-arteritic CRAO improvement has been reported to occur in 22 to 67%; the more benign courses reported in those cases with cilioretinal artery sparing [4]. Various types of treatment rationale for improving energy supply to retinal tissue, e. g. displacement of the embolus, raising retinal perfusion pressure, vasodilatation or increasing blood oxygen content, have been employed in CRAO patients apart from recanalization treatments with intraarterial or intravenous thrombolysis [27]. While none of these approaches has yet been convincingly demonstrated to improve or restore visual acuity, the earlier treatment is initiated, the higher are the odds for a favorable outcome [11]. Homing in on intravenous thrombolysis, existing evidence is equivocal concerning study designs including dosing of tPA, maximally tolerated latency between symptom onset and drug application and study endpoints [11, 28–31]. This impedes a straightforward transfer of existing evidence into guidelines for clinical practice.

In sum, there is a heterogeneity related to early intrahospital management of CRAO including acute diagnostic and therapeutic procedures. While, for example, the guideline of the German Ophthalmological Society ("045/013–S2e-Leitlinie:Retinale arterielle Verschlüsse (RAV)," 2016) stresses the importance of urgent neurological assessment to evaluate vascular risk factors, true interdisciplinary assessment protocols appreciating the specific demands for acute management, e. g. concerning the choice of brain imaging modality or the extent of required laboratory diagnostics, are still lacking.

Strategies are much more uniform when it comes to patient work-up, which is performed by neurologists in more than 75% of hospitals, almost exclusively on SUs. It must be borne in mind, however, that our survey was performed among stroke neurologists, which may bias these results to some extent. Nevertheless, neurological work-up of CRAO patients is essential for several reasons: MRI reveals acute cerebral ischemia in about 30% of CRAO patients, and a relevant proportion of these patients do not suffer further neurological symptoms [32]. Without dedicated imaging of brain parenchyma, ideally by MRI, these patients would be missed, and their increased risk for subsequent stroke [33] would remain un- or underappreciated. In light of the 28-fold increased risk for stroke in the seven days following CRAO [9], close clinical monitoring of patients and the opportunity for timely diagnosis and treatment, should an acute neurological deficit occur, are highly relevant. In a similar vein, the identification of cardiovascular risk factors and the optimization of their treatment are essential components of secondary stroke prevention, which can ideally be accomplished on a SU.

Considering all this, it is somewhat illogical that the work-up of CRAO patients on SUs in Germany is not reimbursed in the same manner as cerebral ischemic stroke. Adding to this inconsistency, amaurosis fugax, i. e. transient loss of vision assumed to be caused by temporary vascular occlusion of a retinal vessel and not associated with permanent deficits, is reimbursed analogously to a transient ischemic attack. As a limitation however, we acknowledge that a questionnaire directed at stroke unit neurologists introduces a certain degree of selection bias, and that addressing ophthalmologists and emergency physicians, who may see CRAO patients at an earlier point in time would have provided additional information. In addition, this study bears limitations inherent to online surveys such as the relatively low response rate (despite several follow-up reminders by e-mail) and the fact that respondents with biases may select themselves into the sample [34, 35].

## Conclusion

**Fig. 2:**
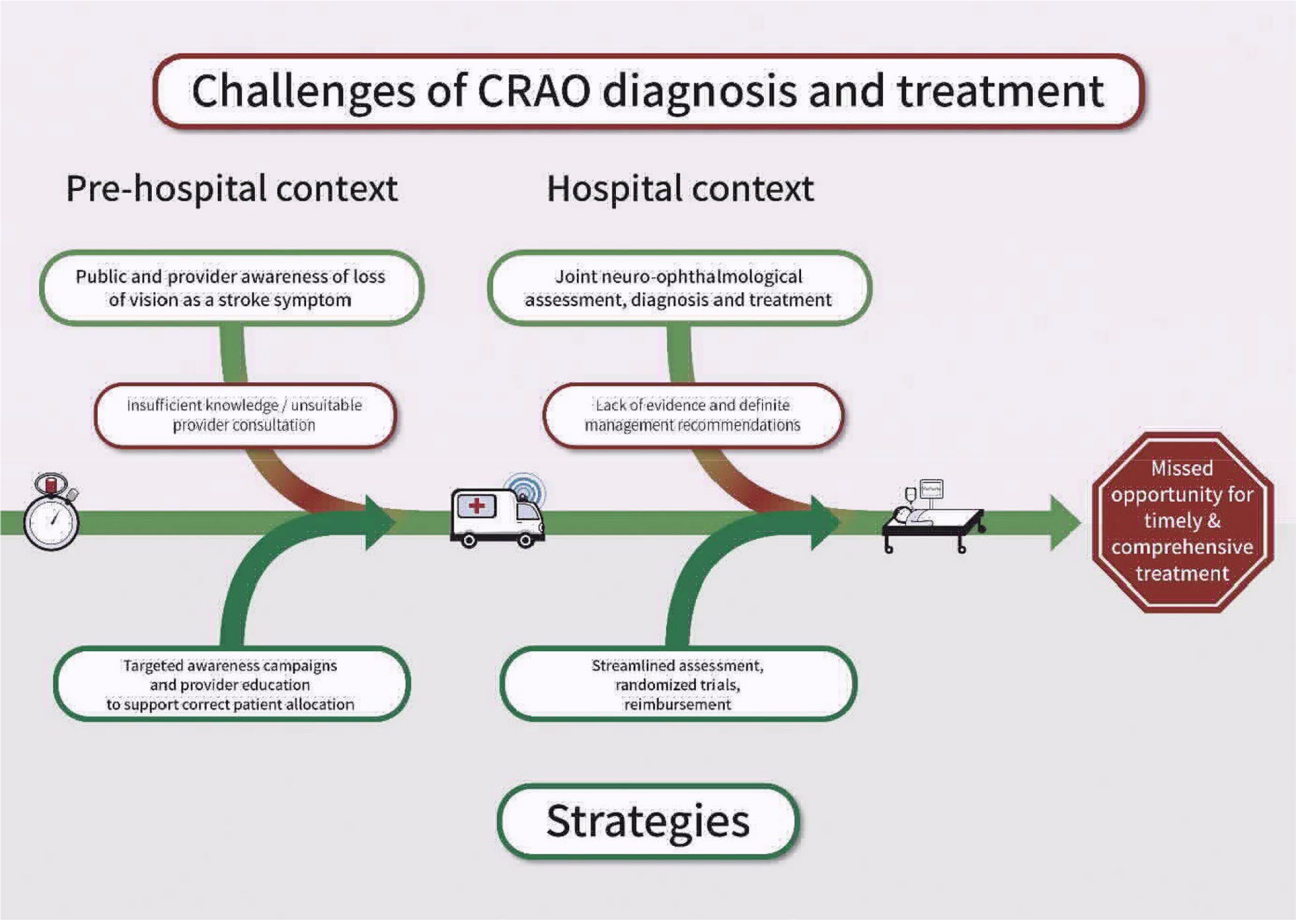
Ichikawa cause-and-effect diagram of the challenges of diagnosis and treatment in patients with central retinal arterial occlusion and suggested approaches to solution

Our findings highlight the challenges related to the swift and comprehensive management of patients with CRAO, which pertain to symptom recognition and patient allocation as well as diagnostic evaluation and treatment (Fig. 2). Hence, while waiting for the results of prospective trials on thrombolysis in CRAO, increasing awareness for UVL as a stroke symptom, streamlining patient disposition to appropriate hospitals and establishing suitable assessment protocols are the major future targets for research as well as public and clinical work.

## Supplementary Information

**Additional file 1.** Original version of the online questionnaire employed in the study.

## Data Availability

The datasets used and/or analyzed during the current study are available from the corresponding author on reasonable request.

## Abbreviations

CRAO: Central retinal artery occlusion
SU: Stroke unit
UVL: Unilateral vision loss
