# Supplementary material for: Current diagnosis and treatment practice of central retinal artery occlusion: results from a survey among German stroke units
Source: Neurol Res Pract. 2022 Aug 1;4:30. doi: 10.1186/s42466-022-00193-w (PMC9341096; doi:10.1186/s42466-022-00193-w)
Supplement: Supplementary file 1 — Additional file 1. Original version of the online questionnaire employed in the study. [file 42466_2022_193_MOESM1_ESM.pdf]

Erfassung der Akutversorgungssituation von PatientInnen mit Zentralarterienverschluss (ZAV) in Neurologischen Kliniken in Deutschland

Die Versorgung von Patienten mit Zentralarterienverschluss ist in Deutschland sehr heterogen organisiert. Um einen Überblick der verschiedenen Behandlungsstrategien und deren Häufigkeit zu gewinnen, erfolgt diese Umfrage.
In dieser Umfrage sind 30 Fragen enthalten.

Versorgungsstruktur

Wird in Ihrer Institution die Behandlung von PatientInnen mit Zentralarterienverschluss standardisiert (*Standardized Operating Procedure* o.ä.) vorgenommen?

*

Bitte wählen Sie nur eine der folgenden Antworten aus:

- ◯ Ja
- ◯ Nein

Werden PatientInnen mit akuter einseitiger schmerzloser Erblindung einer Zentralen Notaufnahme in Ihrer Institution zugewiesen?

*

Bitte wählen Sie nur eine der folgenden Antworten aus:

- ◯ Ja
- ◯ Nein

Anstatt einer zentralen Notaufnahme, wo werden die Patienten stattdessen vorgestellt?

*

Beantworten Sie diese Frage nur, wenn folgende Bedingungen erfüllt sind:
Antwort war 'Nein' bei Frage ' [G01Q02]' (Werden PatientInnen mit akuter einseitiger schmerzloser Erblindung einer Zentralen Notaufnahme in Ihrer Institution zugewiesen?)

❗ Bitte wählen Sie eine der folgenden Antworten:
Bitte wählen Sie nur eine der folgenden Antworten aus:

◯ Augenklinik in eigener Notaufnahme

◯ Sonstiges

Welche Fachrichtung sieht PatientInnen mit akuter einseitiger schmerzloser Erblindung bei Ihnen regulär als erstes? *

❗ Bitte wählen Sie eine der folgenden Antworten:
Bitte wählen Sie nur eine der folgenden Antworten aus:

◯ Neurologie

◯ Augenheilkunde

◯ Sonstiges

Akutdiagnostik

Welche Diagnostik erhalten PatientInnen mit akutem ZAV im Rahmen der Notfallvorstellung **seitens der Augenheilkunde**? *

❗ Bitte wählen Sie die zutreffenden Antworten aus:
Bitte wählen Sie alle zutreffenden Antworten aus:

- [ ] spektrale optische Cohärenz Tomographie (SD-OCT)
- [ ] Neuro-Konsil
- [ ] weiß ich nicht
- [ ] Fundoskopie
- [ ] Keine Augenklinik/kein Augenarzt vor Ort
- [ ] Sonstiges: ____________

Mehrfachnennungen möglich

Welche Diagnostik erhalten PatientInnen mit akutem ZAV im Rahmen der Notfallvorstellung **seitens der Neurologie**? *

❗ Bitte wählen Sie die zutreffenden Antworten aus:
Bitte wählen Sie alle zutreffenden Antworten aus:

- [ ] Bestimmung Blutsenkungsgeschwindigkeit
- [ ] Ultraschall der A. carotis interna
- [ ] MR-Angiographie
- [ ] Magnetresonanztomographie (MRT)
- [ ] Konsil Ophthalmologie
- [ ] Native Computertomographie (CT)
- [ ] CT-Angiographie
- [ ] Sonstiges: ____________

Mehrfachnennung möglich

Therapie

Werden in Ihrer Institution PatientInnen mit akutem ZAV systemisch thrombolysiert? *

Bitte wählen Sie nur eine der folgenden Antworten aus:

- ◯ Ja
- ◯ Nein

In welcher Dosierung und in welchem Zeitfenster verabreichen sie die systemische Lyse? *

Beantworten Sie diese Frage nur, wenn folgende Bedingungen erfüllt sind:
Antwort war 'Ja' bei Frage ' [G03Q01]' (Werden in Ihrer Institution PatientInnen mit akutem ZAV systemisch thrombolysiert?)

Welcher Grund spricht Ihrer Einschätzung nach gegen den Einsatz der Lyse? *

Beantworten Sie diese Frage nur, wenn folgende Bedingungen erfüllt sind:
Antwort war 'Nein' bei Frage ' [G03Q01]' (Werden in Ihrer Institution PatientInnen mit akutem ZAV systemisch thrombolysiert?)

❗ Bitte wählen Sie die zutreffenden Antworten aus:
Bitte wählen Sie alle zutreffenden Antworten aus:

- ☐ fehlende Evidenz
- ☐ Sonstiges: ____________

Mehrfachantwort ist möglich

Werden andere Therapiemöglichkeiten umgesetzt werden **seitens der Neurologie** umgesetzt? *

❗ Bitte wählen Sie die zutreffenden Antworten aus:
Bitte wählen Sie alle zutreffenden Antworten aus:

- [ ] keine der genannten
- [ ] isovolämische Hämodilution
- [ ] Azetazolamid
- [ ] Parazentese
- [ ] hyperbare Sauerstofftherapie
- [ ] Bulbusmassage
- [ ] Sonstiges: ____________

Mehrfachantworten sind möglich!

Werden in Ihrer Institution PatientInnen mit akutem ZAV lokal (i.a.-Therapie) lysiert? *

Bitte wählen Sie nur eine der folgenden Antworten aus:

- ○ Ja
- ○ Nein

Werden andere Therapiemöglichkeiten umgesetzt werden **seitens der Augenheilkunde** umgesetzt? *

❗ Bitte wählen Sie die zutreffenden Antworten aus:
Bitte wählen Sie alle zutreffenden Antworten aus:

- ☐ Bulbusmassage
- ☐ keine der genannten
- ☐ isovolämische Hämodilution
- ☐ Azetazolamid
- ☐ Parazentese
- ☐ hyperbare Sauerstofftherapie
- ☐ Sonstiges: ____________

Mehrfachantworten sind möglich!

Stationäre Weiterversorgung

Werden PatientInnen mit ZAV i.d.R. in die Neurologie aufgenommen? *

❗ Bitte wählen Sie eine der folgenden Antworten:
Bitte wählen Sie nur eine der folgenden Antworten aus:

- ◯ Ja (regulär)
- ◯ Ja (ausschließlich)
- ◯ Nein

Auf welche Station werden die Patienten aufgenommen? *

Beantworten Sie diese Frage nur, wenn folgende Bedingungen erfüllt sind:
Antwort war 'Ja (regulär)' *oder* 'Ja (ausschließlich)' bei Frage ' [G04Q01]' (Werden PatientInnen mit ZAV i.d.R. in die Neurologie aufgenommen?)

❗ Bitte wählen Sie die zutreffenden Antworten aus:
Bitte wählen Sie alle zutreffenden Antworten aus:

- [ ] Stroke Unit
- [ ] Normalstation
- [ ] Intermediate Care

Welche Diagnostik wird im Rahmen der weiteren Abklärung routinemäßig durchgeführt? *

❗ Bitte wählen Sie die zutreffenden Antworten aus:
Bitte wählen Sie alle zutreffenden Antworten aus:

- [ ] CT/MRT falls nicht in der Notaufnahme erfolgt
- [ ] Transösophageale Echokardiografie
- [ ] Transthorakale Echokardiografie
- [ ] Langzeit-EKG
- [ ] Transkranieller Ultraschall
- [ ] Ultraschall der A. carotis interna
- [ ] Sonstiges: ____________________

Mehrfachantworten sind möglich!

Welche sekundärprophlyaktischen Maßnahmen werden routinemäßig ergriffen?

*

❗ Bitte wählen Sie die zutreffenden Antworten aus:
Bitte wählen Sie alle zutreffenden Antworten aus:

- [ ] Keine der genannten
- [ ] Statine
- [ ] orale Antikaogulation unabhängig eines Vorhofflimmerns
- [ ] orale Antikaogulation bei Nachweis eines Vorhofflimmerns
- [ ] Thrombozytenfunktionshemmung
- [ ] Sonstiges: ____________

Mehrfachantworten sind möglich!

Häufigkeit eines ZAV an Ihrem Klinikum

Können Sie angeben, wie viele PatientInnen mit der Diagnose eines ZAV (ICD H.34) in den letzten 2 Jahren in Ihrer Institution **in der Neurologie** stationär behandelt wurden? *

Bitte wählen Sie nur eine der folgenden Antworten aus:

- ◯ Ja
- ◯ Nein

Sowohl Schätzung oder Controllingdaten sind möglich!

Wie gelangen sie an diese Zahlen? *

Beantworten Sie diese Frage nur, wenn folgende Bedingungen erfüllt sind:
Antwort war 'Ja' bei Frage ' [G05Q01]' (Können Sie angeben, wie viele PatientInnen mit der Diagnose eines ZAV (ICD H.34) in den letzten 2 Jahren in Ihrer Institution in der Neurologie stationär behandelt wurden?)

❗ Bitte wählen Sie eine der folgenden Antworten:
Bitte wählen Sie nur eine der folgenden Antworten aus:

◯ Geschätzt

◯ Controlling

◯ Sonstiges [ ]

Wie viele PatientInnen mit der Diagnose eines ZAV (ICD H.34) wurden in den letzten 2 Jahren in Ihrer Institution **in der Neurologie** stationär behandelt? *

Beantworten Sie diese Frage nur, wenn folgende Bedingungen erfüllt sind:
Antwort war 'Ja' bei Frage ' [G05Q01]' (Können Sie angeben, wie viele PatientInnen mit der Diagnose eines ZAV (ICD H.34) in den letzten 2 Jahren in Ihrer Institution in der Neurologie stationär behandelt wurden?)

Können Sie angeben, wie viele PatientInnen mit der Diagnose eines ZAV (ICD H.34) in den letzten 2 Jahren in Ihrer Institution **in der Augenheilkunde** stationär behandelt wurden? *

Bitte wählen Sie nur eine der folgenden Antworten aus:

◯ Ja

◯ Nein

Sowohl Schätzung oder Controllingdaten sind möglich!

Wie gelangen sie an diese Zahlen? *

Beantworten Sie diese Frage nur, wenn folgende Bedingungen erfüllt sind:
Antwort war 'Ja' bei Frage ' [G05Q04]' (Können Sie angeben, wie viele PatientInnen mit der Diagnose eines ZAV (ICD H.34) in den letzten 2 Jahren in Ihrer Institution in der Augenheilkunde stationär behandelt wurden?)

❗ Bitte wählen Sie eine der folgenden Antworten:
Bitte wählen Sie nur eine der folgenden Antworten aus:

◯ Geschätzt

◯ Controlling

◯ Sonstiges [ ]

Wie viele PatientInnen mit der Diagnose eines ZAV (ICD H.34) wurden in den letzten 2 Jahren in Ihrer Institution **in der Augenheilkunde** stationär behandelt? *

Beantworten Sie diese Frage nur, wenn folgende Bedingungen erfüllt sind:
Antwort war 'Ja' bei Frage ' [G05Q04]' (Können Sie angeben, wie viele PatientInnen mit der Diagnose eines ZAV (ICD H.34) in den letzten 2 Jahren in Ihrer Institution in der Augenheilkunde stationär behandelt wurden?)

Fragen zu Ihrer Institution und der Notfallversorgung in Ihrem Haus

Ist Ihre Institution ein universitäres Krankenhaus? *

Bitte wählen Sie nur eine der folgenden Antworten aus:

◯ Ja

◯ Nein

Wie groß ist die Bettenkapazität ihres Hauses? *

Wie ist die Organisation der Notaufnahme in Ihrem Haus? *

❗ Bitte wählen Sie eine der folgenden Antworten:
Bitte wählen Sie nur eine der folgenden Antworten aus:

- ◯ Interdisziplinäre Notaufnahme aller Fachrichtungen
- ◯ Konservative Notaufnahme (neurologisch/internistisch)
- ◯ Eigene Notaufnahmen der Fachrichtungen
- ◯ Sonstiges ____________________

Wie ist die Neurologie in die Notaufnahme integriert? *

❗ Bitte wählen Sie eine der folgenden Antworten:
Bitte wählen Sie nur eine der folgenden Antworten aus:

- ◯ konsiliarisch
- ◯ durch Dienstarzt
- ◯ Sonstiges ____________________

Wie ist die personelle Ausstattung der Notaufnahme, welche primär Patienten mit Zentralarterienverschluss behandelt? *

Bitte geben sie hier nur fest zugeordnete Arztstellen an.

Die Stroke-Unit ihres Hauses ist eine: *

❗ Bitte wählen Sie eine der folgenden Antworten:
Bitte wählen Sie nur eine der folgenden Antworten aus:

◯ telemedizinisch vernetzte Stroke Unit
◯ regionale (Comprehensive) Stroke Unit
◯ überregionale (Comprehensive) Stroke Unit

Abschließende Fragen

Wären Sie bereit, an einer Studie teilzunehmen, die den Effekt der systemischen Lysetherapie mit rt-PA beim akuten Zentralarterienverschluss untersucht? *

❗ Bitte wählen Sie eine der folgenden Antworten:
Bitte wählen Sie nur eine der folgenden Antworten aus:

◯ ja
◯ nein
◯ eventuell

Wir bedanken uns bei Ihnen für die Beantwortung der Fragen. Wenn Sie Anmerkungen zu der Umfrage oder zum Krankheitsbild bzw. der Diagnostik und Therapie haben, wären wir Ihnen dankbar, wenn Sie diese im folgenden Freitext ergänzen. *

Bitte geben Sie Ihre Antwort hier ein:

04.09.2021 – 02:40

Übermittlung Ihres ausgefüllten Fragebogens:
Vielen Dank für die Beantwortung des Fragebogens.
